# Neighboring macrophage-induced alteration in the phenotype of colorectal cancer cells in the tumor budding area

**DOI:** 10.1186/s12935-024-03292-7

**Published:** 2024-03-14

**Authors:** Ichiro Kawamura, Rintaro Ohe, Kazushi Suzuki, Takanobu Kabasawa, Takumi Kitaoka, Daiichiro Takahara, Michihisa Kono, Naoya Uchiyama, Hiroaki Musha, Mitsuru Futakuchi, Fuyuhiko Motoi

**Affiliations:** 1 Department of Surgery I, Yamagata University Faculty of Medicine, Yamagata, Japan; 2 Department of Pathology, Yamagata University Faculty of Medicine, 2-2-2 Iida-Nishi, Yamagata, 990-9585 Japan; 3 Department of Orthopedic Surgery, Yamagata University Faculty of Medicine, Yamagata, Japan

**Keywords:** Colorectal cancer, Tumor budding, Topoisomerase 1, Tumor–stromal interaction, Macrophage, Histological spatial analysis

## Abstract

**Background:**

A higher number of tumor buds in the invasive front of colorectal cancer (CRC) specimens has been shown to contribute to a poor prognosis in CRC patients. Because macrophages (Mφs) have been demonstrated to alter the phenotype of cancer cells, we hypothesized that the phenotype of CRC cells in the tumor budding (TB) area might be changed by the interaction between CRC cells and Mφs.

**Methods:**

We assessed the expression of topoisomerase 1 in CRC cells to estimate the acquisition of chemoresistance in CRC. To demonstrate the tumor–stromal interaction between CRC cells and Mφs, we assessed two histological findings, the number of Mφs per single CRC cell and the proximity between CRC cells and Mφs by histological spatial analysis using HALO software.

**Results:**

The expression levels of topoisomerase 1 in CRC cells were decreased in deeper areas, especially in the TB area, compared to the surface area. Our histological spatial analysis revealed that 2.6 Mφs located within 60 μm of a single CRC cell were required to alter the phenotype of the CRC cell. Double-immunofluorescence staining revealed that higher Mφs were positive for interleukin-6 (IL-6) in the TB area and that AE1/AE3-positive CRC cells were also positive for phospho-STAT3 (pSTAT3) in the TB area; thus, the IL-6 receptor (IL-6R)/STAT3 signaling pathway in CRC cells was upregulated by IL-6 derived from neighboring Mφs.

**Conclusion:**

IL-6 secreted from the neighboring Mφs would alter the phenotype of CRC cells via IL-6R/STAT3 signaling pathway.

**Supplementary Information:**

The online version contains supplementary material available at 10.1186/s12935-024-03292-7.

## Background

Colorectal cancer (CRC) is the second most common cancer and the third leading cause of cancer death worldwide [1]. In the invasive front of CRC specimens, an isolated single cancer cell or a cluster comprising less than five cells is often observed. This lesion has been defined as a tumor bud [2, 3]. Because a higher number of tumor buds in CRC specimens has been shown to be associated with metastatic potential and poor prognosis in CRC patients [4, 5], we assumed that the phenotype of CRC cells in the tumor budding (TB) area may contribute to poor prognosis in CRC patients.

Macrophages (Mφs) have been shown to promote the proliferation of CRC in the tumor microenvironment (TME) [6, 7], and a higher density of Mφs has been shown to be correlated with poor prognosis in CRC patients [6, 7]. Previous study demonstrated that Mφs alter the phenotype of CRC cells in the invasive front to a mesenchymal phenotype (epithelial–mesenchymal transition; EMT) via interleukin-6 (IL-6) [8]. The interaction between CRC cells and Mφs has been shown to induce chemoresistance to 5-fluorouracil [9, 10] and oxaliplatin [11]. Mφs has been shown to induce chemoresistance in CRC cells through the IL-6R/STAT3/miR-204-5p axis [9]. Because Mφs have been shown to alter the phenotype of CRC cells, tumor–stromal interactions between CRC cells and Mφs may be involved in the poor prognosis of CRC patients.

Artificial intelligence has been recently applied to pathological analysis, and machine learning of histopathological images has been shown to be helpful in pathological diagnosis [12]. Additionally, the use of artificial intelligence for predicting lymph node metastasis by whole-slide imaging of CRC specimens has been reported [13]. We previously demonstrated the semiquantitative analysis of stromal cells in immunohistochemistry (IHC) specimens by ImageJ and HALO software (Indica Labs, Albuquerque, NM, USA) using artificial intelligence [14, 15]. These studies demonstrated that the number of stromal cells in the TME would be involved in the malignancy or pathogenesis of diseases [14, 15]. Hence, histological spatial analysis by HALO software may help in examining tumor–stromal interactions between CRC cells and Mφs.

Subpopulations of cancer cells with different phenotypes has shown to assemble and exhibit intratumoral heterogeneity (ITH) [16], which plays important roles in a highly malignant phenotype, characterized by features including chemoresistance [17]. Because topoisomerase 1 (TOPO1) is the enzyme that corrects supercoiled DNA [18, 19], irinotecan, an inhibitor of TOPO1, would be often used as a drug for treating advanced CRC [19]. Irinotecan has also been shown to prevent the proliferation of CRC cells [19]. The decreased expression of TOPO1 in CRC cells has been demonstrated to imply irinotecan resistance [20]. CD205 is an endocytic receptor homologous to the Mφ mannose receptor and is occasionally expressed in cancers [21, 22]. Because the expression of CD205 has been demonstrated to decrease with increasing malignancy in a series of CRC cell lines [23], the low expression of CD205 is considered to correlate with the highly malignant phenotype of CRC cells. Because the expression of TOPO1 and CD205 in CRC cells has been demonstrated to be involved in a malignant phenotype, including chemoresistance, we assumed that TOPO1- and CD205-IHC may reveal the phenotype of CRC cells in the TB area.

We hypothesized that CRC cells in the TB area might acquire a highly malignant phenotype, including chemoresistance, by the interaction between CRC cells and Mφs. We confirmed the difference in overall survival (OS) and relapse-free survival (RFS) based on the number of tumor buds. We also examined the heterogeneity of the expression of TOPO1 and CD205 in CRC specimens and focused on the phenotype of CRC cells in the TB area. To evaluate tumor–stromal interactions between CRC cells and Mφs, histological spatial analysis was used to quantify the number of Mφs per single CRC cell in the TB area and the proximity between CRC cells and Mφs. We examined cytokines derived from Mφs and the activated signaling pathway in CRC cells in the TB area. We also examined the mechanism that cytokine derived from Mφs may alter the phenotype of CRC cells in vitro.

## Methods

### Patients and tissue specimens

One hundred five tissue samples from 105 CRC patients were collected after diagnosis at Yamagata University Hospital between 2010 and 2023. Tissues were fixed with 10% neutral-buffered formalin for 48 h at room temperature and embedded in paraffin. Three-micrometer sections were prepared for hematoxylin–eosin (HE) staining and IHC. The CRC specimens were classified according to the TNM classification (Union for International Cancer Control, 2018). This study was approved by the Research Ethics Committee of Yamagata University Faculty of Medicine (2019-93) and was performed in accordance with the Declaration of Helsinki.

### Evaluation of TB

One hundred tissue samples with accompanying clinical data were used to evaluate TB. The criteria of the International Tumor Budding Consensus Conference 2016 recommend evaluating the number of tumor buds by HE stains [3]. Because the AE1/AE3-IHC specimen has been reported to help in recognizing CRC cells, particularly in TB area [24], we evaluated the number of tumor buds by AE1/AE3-IHC specimens (n = 100) using AE1/AE3 antibody (AE1/AE3, Abcam, Cambridge, UK). The number of tumor buds was confirmed by two board-certified pathologists (OR and FM) based on previous studies [3, 24]. For quantitative analysis of the number of tumor buds, AE1/AE3-IHC specimens were examined under a light microscope and assessed at a magnification of 200× for each specimen. Most of the significant differences in Kaplan‒Meier curves of OS and RFS were observed when the cutoff value was set to 14 buds/field at a magnification of 200×. “TB^high^” was defined as 14 or more buds/field at a magnification of 200×, and “TB^low^” was defined as less than 14 buds/field at a magnification of 200×.

### Definition of depth 1–5

We identified three lesions equidistant from the mucosal surface to the lesion above the invasive front and defined them as depth 1, depth 2, and depth 3. Because the invasive front includes TB, we defined the lesion of the invasive front without TB as depth 4, and we defined the lesion with TB as depth 5.

### IHC studies

Single and double immunostaining were performed with BOND RX^m^ (Leica Biosystems, Nussloch, Germany) according to the manufacturer’s protocol. We used a TOPO1 antibody (EPR5375, Abcam) to estimate the acquisition of chemoresistance in CRC. For quantitative analysis, TOPO1-IHC samples were examined as whole-slide images. The positive ratio of TOPO1 on cancer cells was evaluated at each depth (depth 1–5) of CRC specimens (n = 20). We used a CD205 antibody (LY-75; EPR5233; Abcam) to estimate the malignant potential of CRC. For quantitative analysis, CD205-IHC samples were assessed at a magnification of 200× under a light microscope (n = 100). CD205 positivity was examined in two areas: the invasive front without TB (depth 4) and the TB area (depth 5). CD205-IHC was considered positive if more than 30% of CRC cells were positive. To compare proliferative potential of between CRC cells at depth 4 and that at depth 5, we performed double immunostaining for AE1/AE3 and Ki-67 (K-2, Leica Biosystems) (n = 5). In double immunostaining for AE1/AE3 and Ki-67, three areas were selected from each of the five cases and counted. We used antibodies of CD68 (514H12, Leica Biosystems), CD163 (10D6, Leica Biosystems), CD204 (SRA-E5, Trans Genic Inc, Fukuoka, Japan), and CD206 (5C11, Abnova, Taipei, Taiwan) to evaluate the induction of Mφs in CRC specimens (n = 20). Double immunostaining for CD68 or CD163 and interleukin-6 (IL-6) (1.2-2B11-2G10, Abcam) was performed using the Opal Multiplex IHC KIT (OPAL 4-COLOR AUTOMATION IHC KIT; AKOYA BIOSCIENCES, Marlborough, MA, USA) to estimate Mφ-produced IL-6 (n = 4). Similarly, double immunostaining for AE1/AE3 and phospho-STAT3 (pSTAT3) (D3A7, Cell Signaling Technology, Danvers, Massachusetts) was performed to estimate the upregulation of the IL-6 receptor (IL-6R)/STAT3 pathway in CRC cells (n = 4). An All-in-One Fluorescence Microscope (BZ-X810, KEYENCE, OSAKA, Japan) was used to assess the positivity according to double immunostaining.

### Evaluation of Mφs at each depth in CRC specimens using histological spatial analysis

Whole-slide imaging of the IHC slides of CD68 and CD163 was generated with a virtual scanner (NanoZoomer, HAMAMATSU, Hamamatsu, Japan). To demonstrate the distribution of CD68-positive (CD68^+^) or CD163-positive (CD163^+^) Mφs, histological spatial analysis with HALO software (Indica Labs) was used to allocate positive cells on a grid chart of CD68- or CD163-stained sections. The number of positive cells in a square lesion at each depth of CRC specimens was counted. Histological spatial analysis was used to count the number of cancer cells and Mφs and calculate the ratio of Mφs to surrounding CRC cells at depth 4 and depth 5. Histological spatial analysis identified CD68^+^ Mφs within 30 μm from a single CRC cell with a green color and CD68^+^ Mφs located more than 30 μm from a single CRC cell with a light green color. Furthermore, histological spatial analysis measured the distance between CRC cells and Mφs to evaluate the interaction between CRC cells and CD68^+^ or CD163^+^ Mφs in the selected areas (1 mm^2^) at depth 4 and depth 5, respectively. A histogram was drawn in which the number of CD68^+^ or CD163^+^ Mφs was shown in each class interval of the distance.

### In vitro cell proliferation

The human CRC cell lines, such as HCT116 and SW480, were used in this study. HCT116 were maintained in McCoy’s 5A (Modified) Medium (Thermo Fisher Scientific, Waltham, MA) with 10% fetal bovine serum supplemented with Antibiotic–Antimycotic Mixed Stock Solution (100×) (nacalai tesque, Kyoto, Japan) at 37 °C in a humidified atmosphere containing 5% CO_2_. SW480 were maintained in Leibovitz’s L-15 Medium (Thermo Fisher Scientific) with 10% fetal bovine serum supplemented with Antibiotic–Antimycotic Mixed Stock Solution (100x) (nacalai tesque, Kyoto, Japan) at 37 °C in a humidified atmosphere without CO_2_. HCT116 and SW480 cells were seeded in 96-well plates at the density (5000 cells/well). Following overnight adherence, cells were incubated with medium alone or media containing different concentrations of recombinant IL-6 (0, 0.01 ng/ml) (HumanKine® recombinant human IL-6 protein, Protein Tech, Rosemont, IL) for 24 h. Cell proliferation assay was evaluated by the absorbance value of Cell Counting Kit-8 (CCK-8, DOJINDO, Kumamoto, Japan). SW480 transfected with siRNA against *IL-6 receptor (IL-6R)* (s7314, Thermo Fisher Scientific) by electroporation (Neon Transfection System, Thermo Fisher Scientific), and we established mock cells (SW480^mock^) and *IL-6R* knocked down SW480, SW480^*IL-6R-ND*^ and SW480^*IL-6R-ND2*^. These cell lines were treated with recombinant human IL-6 (HumanKine® recombinant human IL-6 protein, Protein Tech) and were performed cell proliferation assay. All these cell proliferation assays were performed in 10 wells.

### *Quantitative reverse transcription*-*polymerase chain reaction (qRT-PCR)*

qRT-PCR was performed to quantify *IL-6R* mRNA relative to *glyceraldehyde-3-phosphate dehydrogenase (GAPDH)* mRNA. Total RNA was extracted from CRC cell lines (HCT116, SW480, SW480^mock^, SW480^*IL-6R-ND*^, and SW480^*IL-6R-ND2*^) using the ISOGEN system (NIPPON GENE, Tokyo, Japan). The cDNA was synthesized using TaKaRa RNA PCR™ Kit (AMV) Ver.3.0 (Takara, Otsu, Japan). The following primer sequences were designed by Primer3Plus (https://www.primer3plus.com/index.html): 5′-AAACCAATGTTCTCTCTTCTCCAC-3′ and 5′-TATTCCACCCTCTCAAGAGAAAAC-3′ for human *IL-6R* (240 bp). The following primer sequences were used as the internal control: 5′-GCACCGTCAAGGCTGAGAAC-3′ and 5′-TGGTGAAGACGCCAGTGGA-3′ for human *GAPDH* (138 bp) [14]. *IL-6R* and *GAPDH* mRNA levels were assessed using PowerTrack™ SYBR Green Master Mix for qPCR (Thermo Fisher Scientific). Amplification was performed using the QuantStudio3 (Thermo Fisher Scientific). The PCR protocol consisted of initial heat activation at 95 °C for 2 min, followed by 40 cycles of 95 °C for 15 s and 60 °C for 60 s, and dissociation was performed at 95 °C for 15 s, 60 °C for 60 s, and 95 °C for 15 s. The relative gene expression levels were calculated relative to the levels of *GAPDH* mRNA using the comparative Ct (2^−ΔΔCt^) method. All these qRT-PCR experiments were performed in triplicate.

### Statistical analysis

The χ^2^ test, Fisher’s exact test, or the Mann‒Whitney test was used to compare the clinicopathological characteristics of CRC patients. Kaplan‒Meier curves of OS and RFS were drawn and compared by the log-rank test. Univariate and multivariate Cox proportional hazards regression models were used to identify prognostic factors. The Kruskal–Wallis test was used for the TOPO1-positive ratios at all depths. If the ratios were significant, Bonferroni’s multiple comparison test was further applied. The Mann‒Whitney test was used to evaluation the IHC at depth 4 and that of depth 5. The t-test was used to compare relative expression of *IL-6R* mRNA. The Mann‒Whitney test was used to compare absorbance values of CCK-8. Differences with *P* values < 0.05 were considered significant in each analysis. These statistical analyses were performed using JMP version 16 (SAS Institute, Tokyo, Japan).

## Results

### Relationship between the number of tumor buds and prognosis in CRC patients

**Fig. 1:**
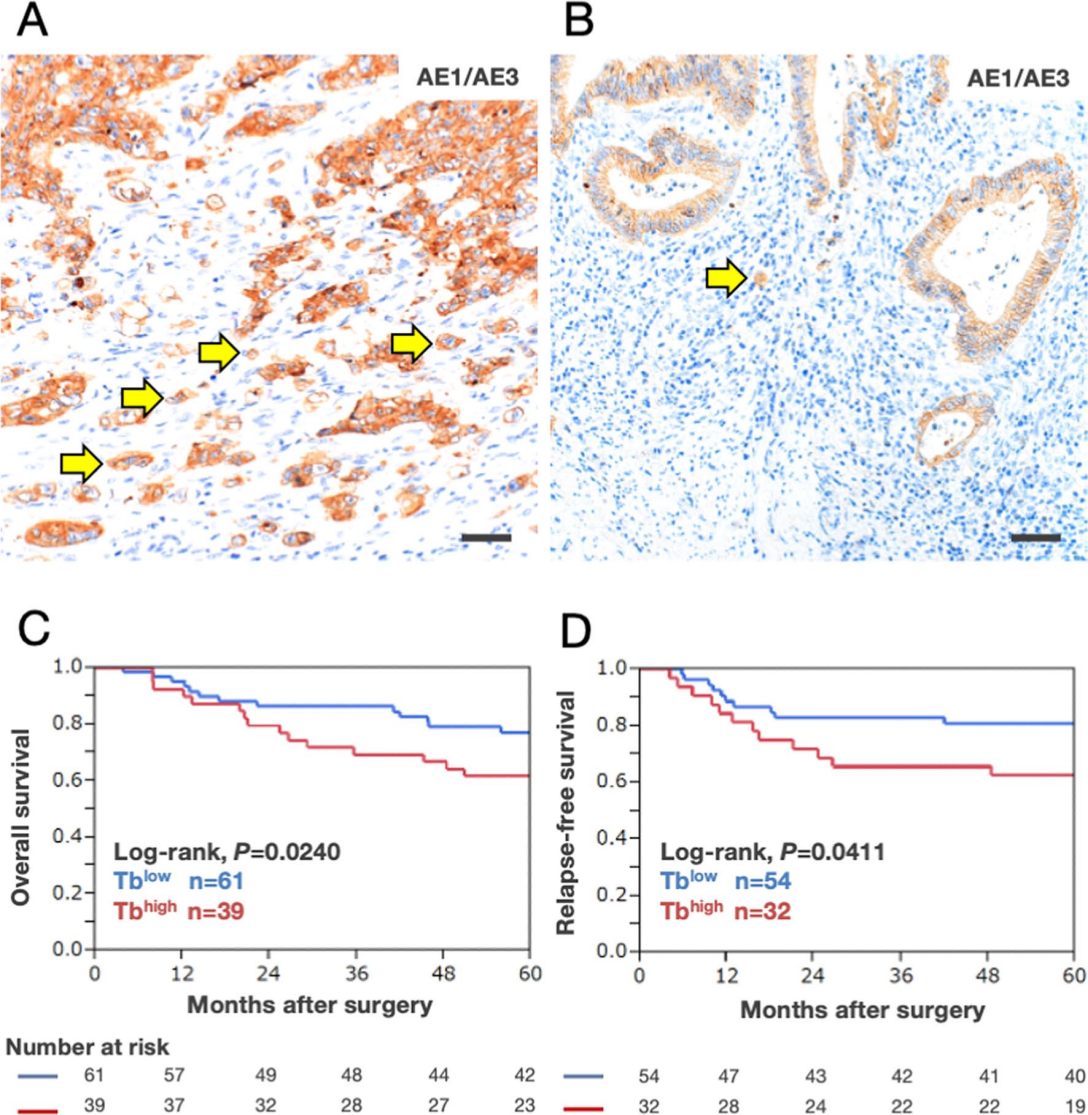
Relationship between the number of tumor buds and prognosis in colorectal cancer patients. **A** Representative AE1/AE3 IHC specimens of CRC cases with high number of tumor budding (TB); yellow arrows indicate isolated CRC cells. Bar, 50 μm. **B** Representative AE1/AE3 IHC specimens of CRC cases with low number of tumor budding (TB); yellow arrows indicate isolated CRC cells. Bar, 50 μm. **C** Kaplan‒Meier curves of overall survival (OS) between TB^high^ and TB^low^. The red curves show the TB^high^ group (n = 39), and the blue curves show the TB^low^ group (n = 61). OS in the TB^high^ group was significantly lower than that in the TB^low^ group (log-rank, *P* = 0.0240). **D** Kaplan‒Meier curves of relapse-free survival (RFS) between TB^high^ and TB^low^. The red curves show the TB^high^ groups (n = 32), and the blue curves show the TB^low^ groups (n = 54). RFS in the TB^high^ group was significantly lower than that in the TB^low^ group (log-rank, *P* = 0.0411)

A typical example of TB was shown in Fig. 1A. To examine the relationship between the number of tumor buds and prognosis in CRC patients, we divided 100 CRC patients into TB^high^ (Fig. 1A) and TB^low^ groups (Fig. 1B). Kaplan‒Meier analysis revealed a statistically significant difference in OS (Fig. 1C) and RFS (Fig. 1D), including all stages (I-IV), when the cutoff value of the number of tumor buds was set to 14 at a magnification of 200×. At week 60, OS in the TB^high^ group was significantly lower than that in the TB^low^ group (Fig. 1C). Similarly, RFS in the TB^high^ group was significantly lower than that in the TB^low^ group (Fig. 1D). No significant difference was found in OS and RFS between the TB^high^ group and the TB^low^ group with matched patient backgrounds (stages I, II, III, or IV). Except for the number of tumor buds, no significant differences were demonstrated between the two groups in clinicopathological characteristics (Additional file 2: Table S1). Univariate analyses of OS and RFS revealed that poor prognosis was correlated with TB^high^ by Cox proportional hazards regression models (Additional file 3: Table S2). Multivariate analyses on OS and RFS revealed that only stage III/IV was an independent poor prognostic factor. Our results indicated that the number of tumor buds was one of the prognostic factors in CRC patients.

### Heterogeneity of TOPO1 expression in CRC specimens at each depth

**Fig. 2:**
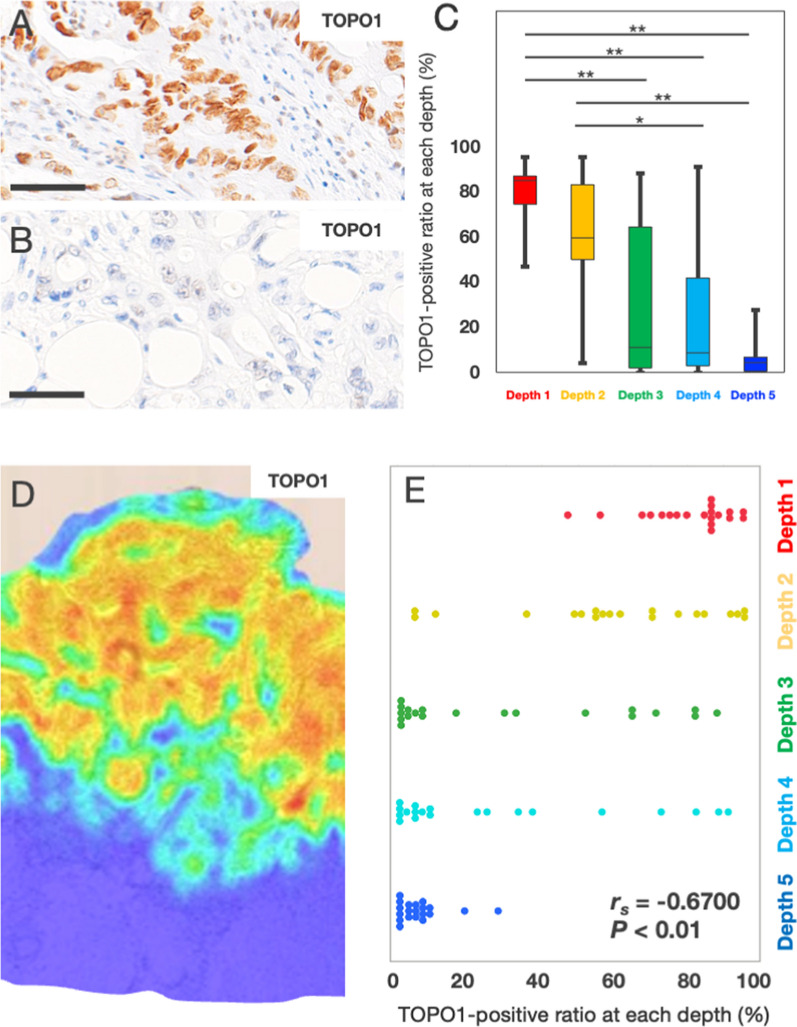
Heterogeneity of topoisomerase 1 expression in colorectal cancer specimens at each depth. **A** Representative topoisomerase 1 (TOPO1) immunohistochemistry in colorectal cancer (CRC) in the mucosal surface area (depth 1); 98.5% of CRC cells were positive for TOPO1 (134/136 cells) in this case. Bar, 50 μm. **B** Representative topoisomerase 1 (TOPO1) immunohistochemistry in colorectal cancer (CRC) in the TB area (depth 5); No cells were positive for TOPO1 in this case. Bar, 50 μm. **C** Heterogeneity of the positive ratio of TOPO1 in CRC cells at each depth; Boxplots showed that the TOPO1-positive ratios at depth 1, 2, 3, 4, and 5 were 80.3 ± 12.7%, 60.0 ± 28.8%, 30.4 ± 33.4%, 27.5 ± 32.6%, and 5.7 ± 6.7%, respectively (n = 20, *P < 0.05, **P < 0.01). The statistically significant difference was observed between depth 1 and 3, 1 and 4, 1 and 5, 2 and 4, and 2 and 5. **D** Representative heatmap of TOPO1-IHC; The red to yellow colored area indicates a higher density of TOPO1-positive cells in the mucosal area (depth 1–3). The green to blue colored area indicates a lower density of TOPO1-positive cells in the invasive front without TB (depth 4) and TB area (depth 5). **E** Correlation between the positive ratio of TOPO1 in CRC cells and the depth of CRC specimens; the TOPO1-positive ratio was negatively correlated with depth by scatter plot analysis (n = 20, *r*_*s*_ = − 0.6700, *P* < 0.01)

We observed TOPO1-positive findings in the nuclei of CRC cells (Fig. 2A). Among the TB^high^ cases, 20 cases were selected to elucidate the heterogeneity of TOPO1 positivity. We examined the expression of TOPO1 based on the depth of CRC specimens. The representative TOPO1-IHC showed that 98.5% of CRC cells were positive at depth 1 (Fig. 2A) and no CRC positive cells at depth 5 (Fig. 2B). Quantitative analysis revealed that the TOPO1-positive ratios at depth 1, 2, 3, 4, and 5 were 80.3 ± 12.7%, 60.0 ± 28.8%, 30.4 ± 33.4%, 27.5 ± 32.6%, and 5.7 ± 6.7%, respectively (n = 20) (Fig. 2C). The representative heatmap of the TOPO1-positive cells revealed that a higher density was observed in the mucosal area (depths 1–3), a lower density in the invasive front (depth 4), and a significantly lower density in the TB area (depth 5) (Fig. 2D). Furthermore, scatter plot between the positivity of TOPO1 and the depth revealed that the low TOPO1-positive ratio was negatively correlated with deeper depth (n = 20, *r*_*s*_ = − 0.6700, *P* < 0.01) (Fig. 2E). These results indicated the heterogeneity of TOPO1 positivity in CRC specimens, and the TOPO1-positive ratio was lowest in the TB area (depth 5).

### Comparison of the phenotype of CRC cells at depth 4 and depth 5

**Fig. 3:**
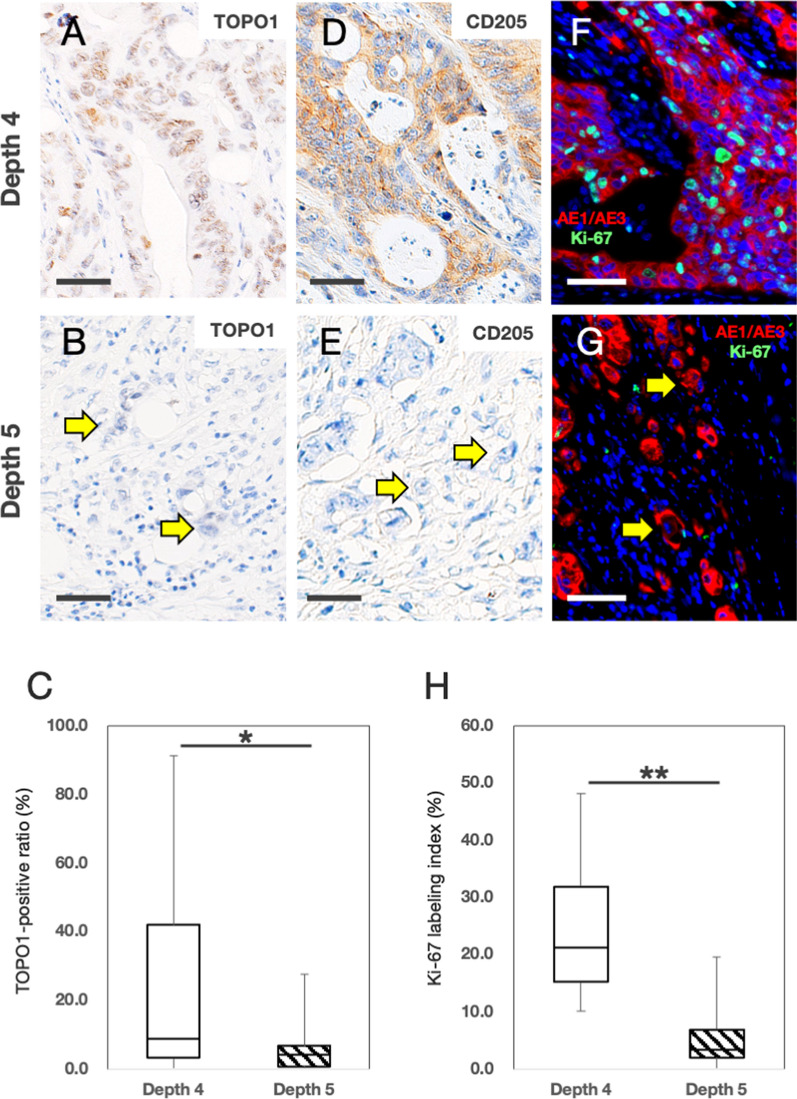
Comparison of the phenotype of colorectal cancer (CRC) cells at depth 4 and depth 5. **A**, **B** Representative topoisomerase 1 (TOPO1)-immunohistochemistry (IHC) in CRC at depth 4 (**A**) and depth 5 (**B**). 90.6% TOPO1-positive (TOPO1^+^) CRC cells proliferated at a depth of 4 (**A**, 164 TOPO1^+^ cells/181 CRC cells). Tumor buds were negative for TOPO1 at depth 5 (**B**, yellow arrows). **C** Boxplot of TOPO1 positivity in CRC cells at depth 4 and depth 5 (n = 20). In TOPO1-IHC, two areas (depth 4 and depth 5) were selected from each of the twenty cases and counted. The TOPO1 positivity rate in CRC cells at depth 5 was lower than that at depth 4 (depth 4, 27.5 ± 32.6%; depth 5, 5.7 ± 6.7%; *, *P* < 0.05). **D**, **E** Representative CD205-IHC in CRC at depth 4 (**D**) and depth 5 (**E**). 95.9% CD205-positive CRC cells proliferated at a depth of 4 (**D**, 186 CD205^+^ cells/194 CRC cells). Tumor buds were negative for CD205 at depth 5 (**E**, yellow arrows). **F**, **G** Representative double immunostaining for AE1/AE3 and Ki-67 in CRC at depth 4 (**F**) and depth 5 (**G**). 43.8% Ki-67-positive CRC cells proliferated at a depth of 4 (**F**, 92 Ki-67^+^ cells/210 AE1/AE3^+^ cells). Tumor buds were negative for Ki-67 at depth 5 (**G**, yellow arrows). **H** Boxplot of Ki-67 positivity in CRC cells at depth 4 and depth 5 (n = 5). In double immunostaining for AE1/AE3 and Ki-67, three areas were selected from each of the five cases and counted. The Ki-67 positivity rate in CRC cells at depth 5 was lower than that at depth 4 (depth 4, 24.2 ± 12.1%; depth 5, 5.3 ± 5.3%; **P < 0.01). Bars, 50 μm

To emphasize the difference in the phenotype of CRC cells at depth 4 and 5, we compared the TOPO1-IHC positive findings of CRC cells at depth 4 and 5. The TOPO1-positive ratios at depth 5 were statistically lower than those at depth 4 (depth 4, 27.5 ± 32.6%; depth 5, 5.7 ± 6.7%; *P* = 0.049) (Fig. 3A–C). We compared the expression of CD205 in CRC cells at depth 4 and 5 by IHC. In 14 of CD205 positive cases, the positive ratio of CD205 was 86.7 ± 14.4% at depth 4 (Fig. 3D), no CD205-positive CRC cells were observed at depth 5. (Fig. 3E). In 86 of CD205 negative cases, no CD205 positive cells were observed at depth 4 and 5. Furthermore, we compared the Ki-67 labeling indices (LI) of CRC cells at depth 4 and 5 to evaluate the proliferative potential. We found that the Ki-67 LI of CRC cells at depth 5 were statistically lower than those at depth 4 (Fig. 3F–H; depth 4, 24.2 ± 12.1%; depth 5, 5.3 ± 5.3%; *P* < 0.01). These results indicated that the phenotype of CRC cells at depth 5 (the TB area) was different from that at depth 4 (the invasive front without TB).

### Number of Mφs per single CRC cell at depth 4 and depth 5

**Fig. 4:**
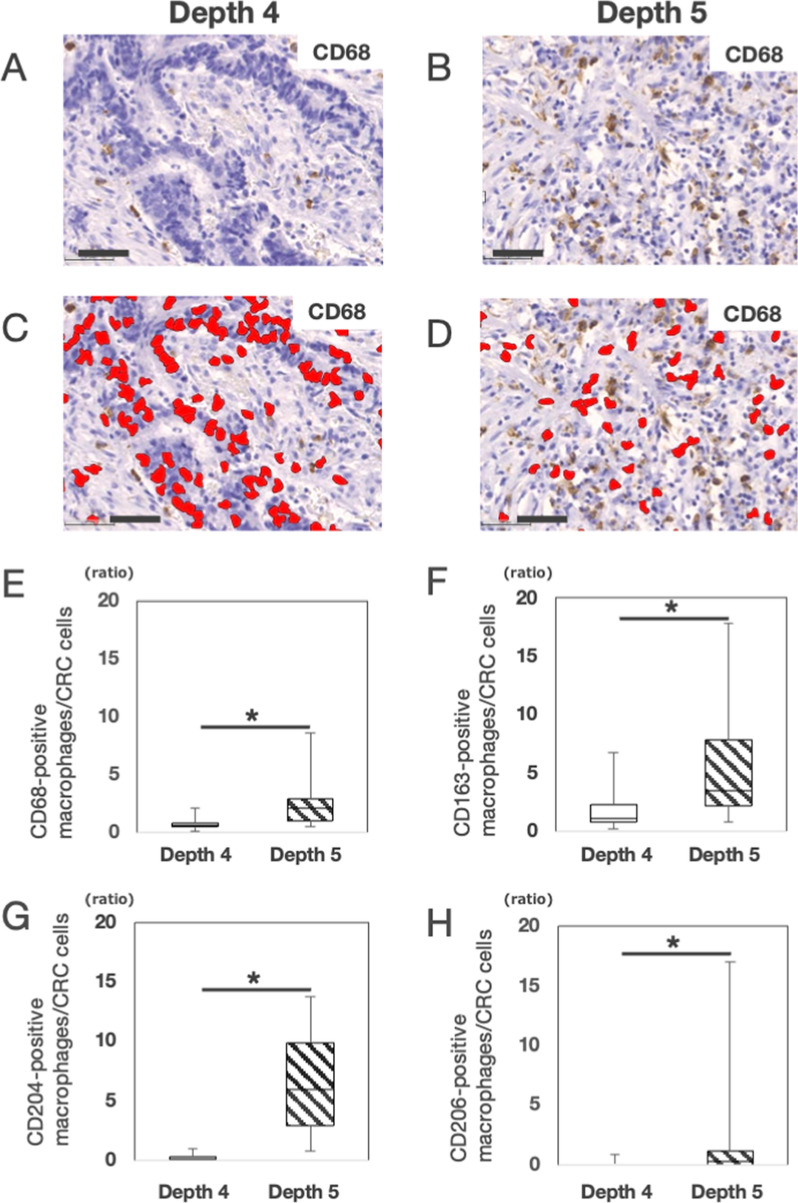
Number of macrophages per single colorectal cancer cell at depth 4 and depth 5. **A** The representative CD68-immunohistochemistry (IHC) in colorectal cancer (CRC) at depth 4; Eight CD68-positive (CD68^+^) macrophages (Mφs) infiltrated to depth 4 in this case. Bar, 50 μm. **B** The representative CD68-immunohistochemistry (IHC) in colorectal cancer (CRC) at depth 5; Fifty CD68^+^ Mφs infiltrated to a depth of 5 in this case. Bar, 50 μm. **C** Histological spatial analysis identified CRC cells as a red color at depth 4; One hundred twenty-five CRC cells were observed at depth 4 in this case. Bar, 50 μm. **D** Histological spatial analysis identified CRC cells as a red color at depth 5; Forty CRC cells were observed at depth 5 in this case. Bar, 50 μm. **E** Comparison between the number of CD68^+^Mφs per single CRC cell at depth 4 and that at depth 5; based on the representative figure **A** and **C**, the number of CD68^+^ Mφs per single CRC cell at depth 4 was calculated to be 0.064 (8/125) in this case., and the mean ± SD in 20 cases was 0.69 ± 0.45. Based on the representative figure **B** and **D**, the number of CD68^+^ Mφs per single CRC cell at depth 5 was calculated to be 1.25 (50/40) in this case, and the mean ± SD in 20 cases was 2.56 ± 2.19. the Boxplot showed that the number of CD68^+^Mφs per single CRC cell at depth 5 was statistically higher than that at depth 4 (*P* < 0.01). **F** Comparison between the number of CD163^+^Mφs per single CRC cell at depth 4 and that at depth 5; the Boxplot showed that the number of CD163^+^Mφs per single CRC cell at depth 5 was statistically higher than that at depth 4 (n = 20; depth 4, 1.78 ± 1.57; depth 5, 5.31 ± 4.30; *P* < 0.01). **G** Comparison between the number of CD204^+^Mφs per single CRC cell at depth 4 and that at depth 5; the Boxplot showed that the number of CD204^+^Mφs per single CRC cell at depth 5 was statistically higher than that at depth 4 (n = 20; depth 4, 0.22 ± 0.29; depth 5, 6.70 ± 4.48; *P* < 0.01). **H** Comparison between the number of CD206^+^Mφs per single CRC cell at depth 4 and that at depth 5; the Boxplot showed that the number of CD206^+^Mφs per single CRC cell at depth 5 was statistically higher than that at depth 4 (n = 20; depth 4, 0.10 ± 0.24; depth 5, 1.34 ± 3.74; *P* < 0.01)

Because Mφs have been shown to alter the phenotype of cancer cells through tumor–stromal interactions, we hypothesized that Mφs may alter the phenotype of CRC cells in the TB area (depth 5). A higher number of Mφs near CRC cells would be involved in tumor–stromal interactions at depth 5; we compared the number of Mφs at depth 5 in 20 cases with that at depth 4 in the same cases. In histological spatial analysis of the representative case (Fig. 4A, B), we found that eight CD68^+^ Mφs infiltrated at depth 4 (Fig. 4A) and 50 Mφs infiltrated at depth 5 (Fig. 4B). Histological spatial analysis identified the nuclei of CRC cells and allocated CRC cells with a red color (Fig. 4C, D) on a grid chart. One hundred twenty-five CRC cells integrated into cancer tissue at depth 4 (Fig. 4C), and 40 isolated CRC cells were observed at depth 5 (Fig. 4D). The number of CD68^+^ Mφs per single CRC cell at depth 4 was 0.69 ± 0.45, and that at depth 5 was 2.56 ± 2.19. Similarly, the number of CD163^+^ Mφs per single CRC cell at depth 4 was 1.78 ± 1.57, and that at depth 5 was 5.31 ± 4.30. The number of CD204^+^ Mφs per single CRC cell at depth 4 was 0.22 ± 0.29, and that at depth 5 was 6.70 ± 4.48. The number of CD206^+^ Mφs per single CRC cell at depth 4 was 0.10 ± 0.24, and that at depth 5 was 1.34 ± 3.74. The number of Mφs per single CRC cell at depth 5 was higher than that at depth 4 in all the Mφ markers (*P* < 0.01) (Fig. 4E–H). In M2 makers, the number of CD206^+^ Mφs at depth 5 was very small compared to those of CD163^+^ Mφs and CD204^+^ Mφs (*P* < 0.01).

### Proximity between CRC cells and Mφs

**Fig. 5:**
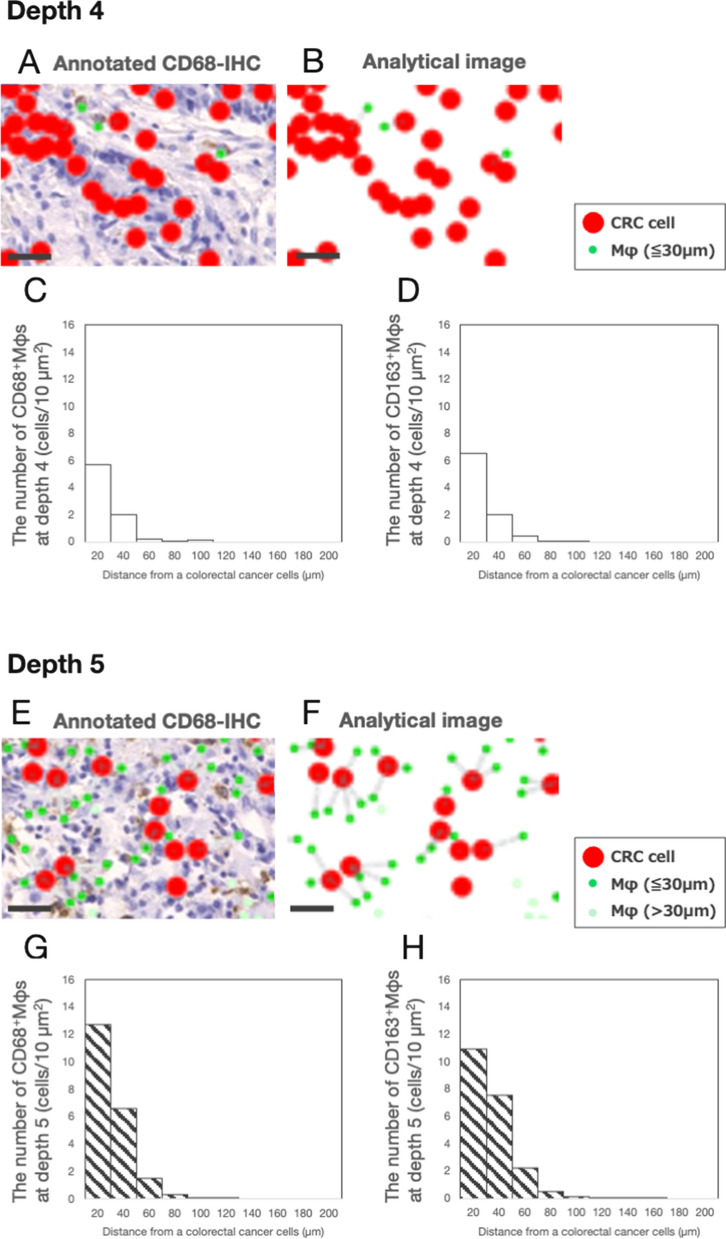
Proximity between colorectal cancer cells and macrophages. **A** The representative CD68 immunohistochemistry-(IHC) in colorectal cancer (CRC) at depth 4; To evaluate tumor–stromal interactions between CRC cells and macrophages (Mφs), CRC cells and CD68-positive (CD68^+^) Mφs were annotated. The representative image of CD68-IHC identified 33 CRC cells as red dots and three CD68^+^ Mφs as green dots at depth 4 by HALO software. Bar, 30 μm. **B** Histological spatial analysis clipped the colored dots of **A**; Thirty-three red dots and three green dots at depth 4 were clipped and the extracted images (analytical images) could clarify the positioning of these dots. The gray-colored lines show the shortest distance between a single CRC cell (red dots) and CD68^+^ Mφs within 30 µm of the CRC cell (green dots). Bar, 30 μm. **C** The number of CD68^+^ Mφs in each interval of the distance between CRC cells and CD68^+^ Mφs at depth 4; The histogram revealed that 98.3% of CD68^+^Mφs were accumulated within 60 μm. The highest accumulation of CD68^+^Mφs was observed between 0 and 20 μm. **D** The number of CD163^+^ Mφs in each interval of the distance between CRC cells and CD163^+^ Mφs at depth 4; The histogram revealed that 99.5% of CD163^+^Mφs were accumulated within 60 μm. The highest accumulation of CD163^+^Mφs was observed between 0 and 20 μm. **E** The representative CD68-IHC in CRC at depth 5; similar to **A**, CRC cells and CD68-positive (CD68^+^) Mφs were annotated. The representative image of CD68-IHC identified 13 CRC cells as red dots and 37 CD68^+^ Mφs as green and light green dots at depth 5 by HALO software. Bar, 30 μm. **F** Histological spatial analysis clipped the colored dots from **E**; Thirteen red dots and 37 green and light green dots at depth 5 were clipped and the extracted images (analytical images) could clarify the positioning of these dots. The gray-colored lines show the shortest distance between a single CRC cell (red dots) and CD68^+^ Mφs within 30 µm of the CRC cell (green dots). Bar, 30 μm. **G** The number of CD68^+^ Mφs in each interval of the distance between CRC cells and CD68^+^ Mφs at depth 5; the histogram revealed that 98.2% of CD68^+^Mφs were accumulated within 60 μm. The highest accumulation of CD68^+^Mφs was observed between 0 and 20 μm. **H** The number of CD163^+^ Mφs in each interval of the distance between CRC cells and CD163^+^ Mφs at depth 5; The histogram revealed that 96.7% of CD163^+^Mφs were accumulated within 60 μm. The highest accumulation of CD163^+^Mφs was observed between 0 and 20 μm

To estimate the proximity between CRC cells and Mφs, we compared the distance between CRC cells and Mφs in the invasive front without TB (depth 4) with that in the TB area (depth 5). The representative image of CD68-IHC identified 33 CRC cells as red dots and three CD68^+^ Mφs as green dots at depth 4 by HALO software (Fig. 5A). Histological spatial analysis clipped the colored dots from Fig. 5A and these extracted images could clarify the positioning of these colored dots (Fig. 5B). The histogram revealed that 98.3% of CD68^+^Mφs accumulated within 60 μm at depth 4 (Fig. 5C). Similar results were obtained by CD163-IHC (depth 4, 99.5%) (Fig. 5D). The highest accumulation of CD68^+^Mφs and CD163^+^Mφs was observed between 0 and 20 μm (Fig. 5C, D).

The representative image of CD68-IHC identified 13 CRC cells as red dots and 37 CD68^+^ Mφs as green and light green dots at depth 5 by HALO software (Fig. 5E). Histological spatial analysis clipped the colored dots from Fig. 5E and these extracted images could clarify the positioning of these colored dots (Fig. 5F). The histogram revealed that 98.2% of CD68^+^Mφs accumulated within 60 μm at depth 5 (Fig. 5G). Similar results were obtained by CD163-IHC (depth 5, 96.7%) (Fig. 5H). The highest accumulation of CD68^+^Mφs and CD163^+^Mφs was observed between 0 and 20 μm (Fig. 5G, H).

Thus, we found that a higher number of neighboring CD68^+^Mφs and CD163^+^Mφs were located within 60 µm surrounding CRC cells in the TB area (depth 5) than in the invasive margin without TB (depth 4). Our histological spatial analysis indicated that neighboring Mφs interact with CRC cells.

### Upregulation of the IL-6R/STAT3 pathway in CRC cells in the TB area by IL-6 derived from neighboring Mφs

**Fig. 6:**
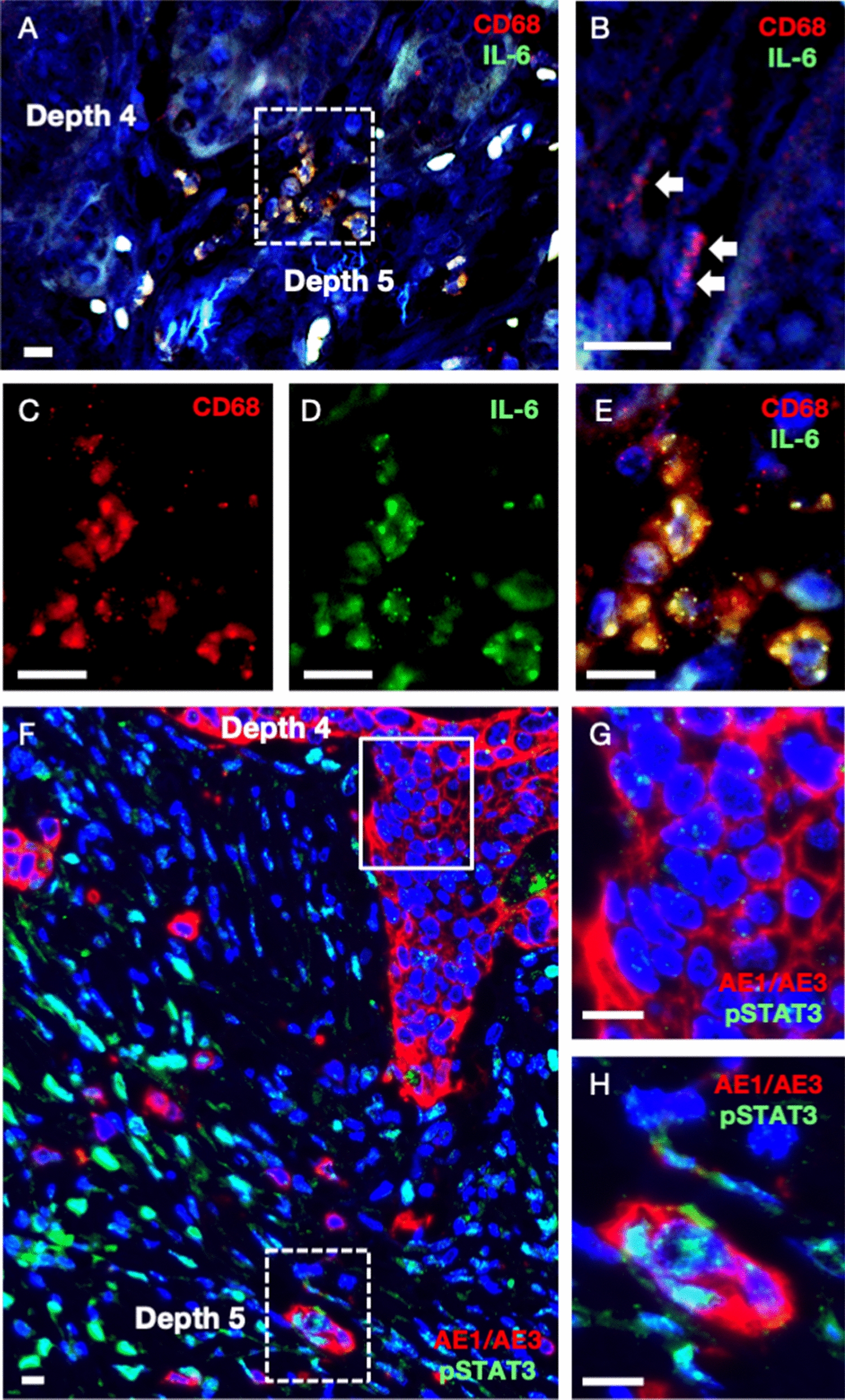
Upregulation of the IL-6R/STAT3 pathway in colorectal cancer cells in the tumor budding area by IL-6 derived from neighboring macrophages. **A**–**D** The representative double immunofluorescence of CD68 and IL-6. **A** A low magnification of double immunofluorescence was shown at depth 4 and depth 5. Nuclear staining was performed with DAPI. **B** In a high-power field at depth 4, CD68-positive and IL-6-negative macrophages were labeled with TRITC red (arrows). The dashed-line rectangle at depth 5 is magnified in **C**–**E**. **C** CD68-positive macrophages were labeled with TRITC red. **D** IL-6-positive cells were labeled with FITC green. **E** The merged yellow was observed at depth 5. **F**–**H** The representative double immunofluorescence of AE1/AE3 and phospho-STAT3 (pSTAT3). **F** A low magnification of double immunofluorescence was shown at depth 4 and depth 5. Nuclear staining was performed with DAPI. The solid line rectangle at depth 4 is magnified in (**G**). **G** In a high-power field at depth 4, AE1/AE3-positive and IL-6-negative colorectal cancer cells were labeled with TRITC red. The dashed-line rectangle at depth 5 is magnified in (**H**). **H** AE1/AE3-positive cytoplasm of colorectal cancer cells were labeled with TRITC red. pSTAT3-positive nuclear of the cell was labeled with FITC green. The AE1/AE3-positive and pSTAT3-positive cell was observed at depth 5. These double immunofluorescences were performed on four samples. Bars, 10 μm

To identify cytokine involvement from Mφs in CRC specimens, we preliminarily examined IHC studies of IL-6, IL-8, and C-C motif chemokine ligand 2 (CCL2). IL-6 was successfully stained with Mφs. Representative images of double immunofluorescence of CD68 and IL-6 was shown in Fig. 6A. At higher magnification of the invasive margin without TB (depth 4), CD68^+^ cells were negative for IL-6 (Fig. 6B). At higher magnification of the TB area (depth 5), CD68^+^ Mφs were labeled with TRITC red (Fig. 6C), and IL-6-positive cells were labeled with FITC green (Fig. 6D). The merged yellow staining demonstrated that most CD68^+^ cells were positive for IL-6 (Fig. 6E). Similar results were obtained by double immunofluorescence of CD163 and IL-6 (Additional file 1: Fig. S1). These results indicated that IL-6 derived from CD68^+^ and CD163^+^ Mφs was involved in tumor–stromal interactions in the TB area.

To demonstrate the activation of the IL-6R/STAT3 pathway in CRC cells in the TB area, representative images of double immunofluorescence of AE1/AE3 and pSTAT3 was shown in Fig. 6F. At a higher magnification of the invasive margin without TB (depth 4), AE1/AE3-positive CRC cells were negative for pSTAT3 (Fig. 6G). At higher magnification of the TB area (depth 5), AE1/AE3-positive and pSTAT3-positive CRC cells were observed (Fig. 6H). Taken together, IL-6 derived from Mφs may activate the IL-6R/STAT3 pathway in CRC cells in the TB area.

### Expressions of IL-6R mRNA and cell proliferation assay in CRC cell lines

**Fig. 7:**
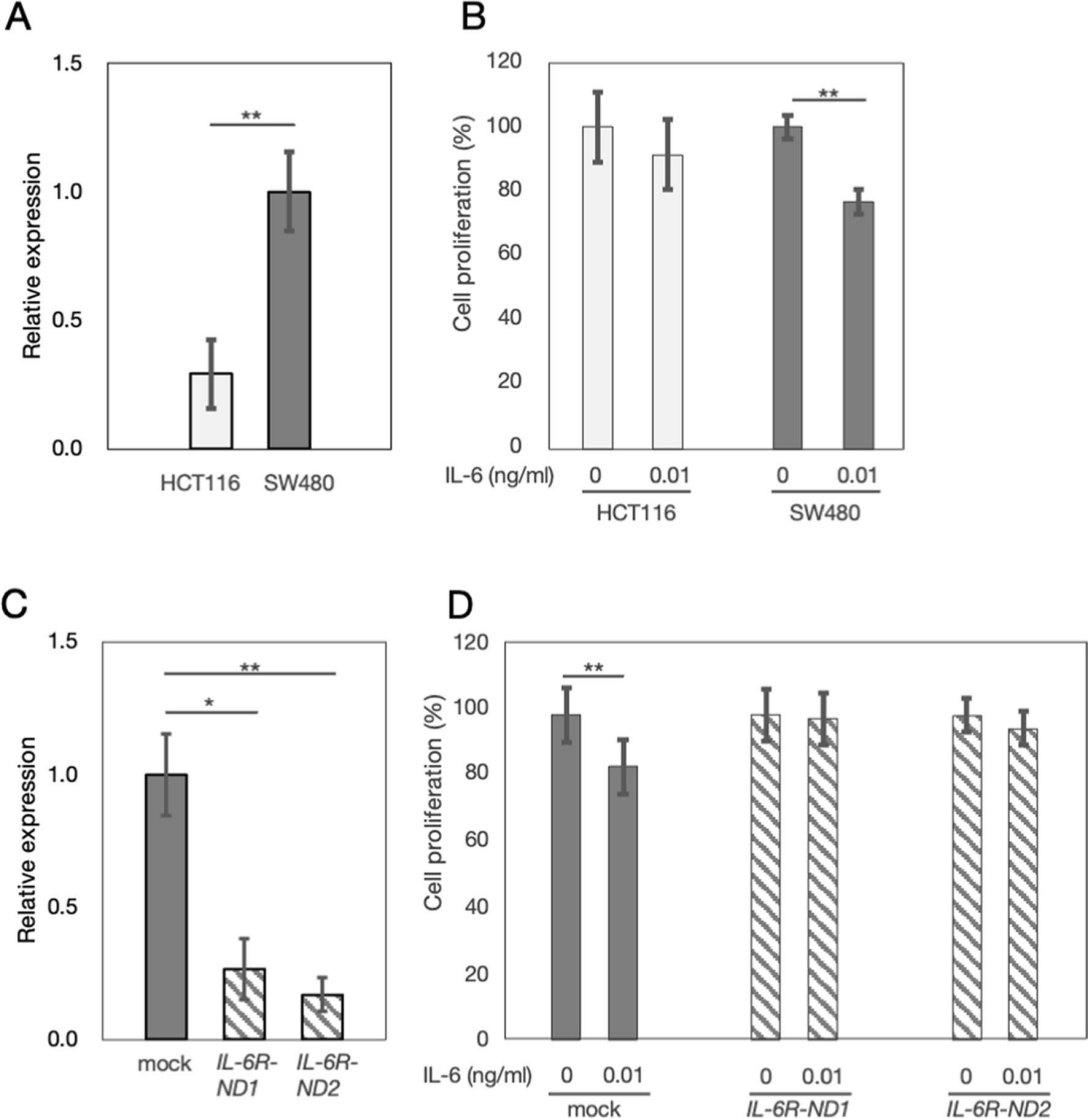
Expressions of IL-6 receptor (IL-6R) mRNA and cell proliferation assay in colorectal cancer cell lines. **A** *IL-6R* mRNA expression of relative to *glyceraldehyde-3-phosphate dehydrogenase* (*GAPDH*) mRNA expression in colorectal cancer cell lines HCT116 and SW480. The mRNA expression of *IL-6R* was significantly lower in HCT116 than that in SW480 (Relative expression of *IL-6R*; HCT116, 0.29 ± 0.13; SW480, 1.00 ± 0.16; *P* < 0.01). For relative expression, the mean value of SW480 was set to 1. **B** Effect of recombinant IL-6 on HCT116 and SW480. Treatment of recombinant IL-6 suppressed the proliferation of SW480 cells (absorbance value of CCK-8; IL-6 0 ng/ml, 100 ± 3.7%; IL-6 0.01 ng/ml, 77 ± 2.4%; *P* < 0.01), although the treatment did not suppress the proliferation of HCT116 cells (absorbance value of CCK-8; IL-6 0 ng/ml, 100 ± 11.2%; IL-6 0.01 ng/ml, 91 ± 10.9%). **C** *IL-6R* mRNA expression in mock cells (SW480^mock^), and IL-6R knocked down cell (SW480^*IL-6R-ND1*^, and SW480^*IL-6R-ND2*^) mRNA expressions of *IL-6R* in SW480^*IL-6R-ND1*^ and SW480^*IL-6R-ND2*^ were significantly lower than that in mock cells (SW480^mock^) (Relative expression of *IL-6R*; SW480^mock^, 1.00 ± 0.15; SW480^*IL-6R-ND1*^, 0.27 ± 0.11, *P* < 0.05; SW480^*IL-6R-ND2*^, 0.17 ± 0.06, *P* < 0.01). For relative expression, the mean value of SW480^mock^ was set to 1. **D** Effect of recombinant IL-6 on mock cells (SW480^mock^), and IL-6R knocked down cell (SW480^*IL-6R-ND1*^, and SW480^*IL-6R-ND2*^). Treatment of recombinant IL-6 did not suppress proliferation of SW480^*IL-6R-ND1*^ (Absorbance value of CCK-8; IL-6 0 ng/ml, 100 ± 8.0%; IL-6 0.01 ng/ml, 99 ± 10.1%) and SW480^*IL-6R-ND2*^ (Absorbance value of CCK-8; IL-6 0 ng/ml, 100 ± 4.65%; IL-6 0.01 ng/ml, 90 ± 4.4%), although the treatment suppressed proliferation of SW480^mock^ (Absorbance value of CCK-8; IL-6 0 ng/ml, 100 ± 9.3%; IL-6 0.01 ng/ml, 84 ± 7.6%; *P* < 0.01). All these qRT-PCR experiments were performed in triplicate and all these cell proliferation assays were performed in 10 wells. *P < 0.05, **P < 0.01

To investigate altering the phenotype of CRC cells via IL-6/IL-6R, we chose two CRC cell lines, HCT116 and SW480, with different expression of *IL-6R* based on CellExpress (http://cellexpress.cgm.ntu.edu.tw/) [25]. We found mRNA expression of *IL-6R* in SW480 was higher than that in HCT116 (Relative expression of *IL-6R*; SW480, 1.00 ± 0.16; HCT116, 0.29 ± 0.13; *P* < 0.01) (Fig. 7A). Cell proliferation assay revealed that treatment of recombinant IL-6 suppressed the proliferation of SW480 cells (Absorbance value of CCK-8; IL-6 0 ng/ml, 100 ± 3.7%; IL-6 0.01 ng/ml, 77 ± 2.4%; *P* < 0.01), although that treatment did not suppress the proliferation of HCT116 cells (Absorbance value of CCK-8; IL-6 0 ng/ml, 100 ± 11.2%; IL-6 0.01 ng/ml, 91 ± 10.9%) (Fig. 7B). These results indicated that IL-6/IL-6R may suppress proliferation of SW480 with high-expression of IL-6R.

To confirm that involvement of IL-6/IL-6R on proliferation of SW480, we established SW480^*IL-6R-ND1*^ and SW480^*IL-6R-ND2*^. The mRNA expressions of *IL-6R* in SW480^*IL-6R-ND1*^ and SW480^*IL-6R-ND2*^ were lower than that in mock cells (SW480^mock^) (Relative expression of *IL-6R*; SW480^mock^, 1.00 ± 0.15; SW480^*IL-6R-ND1*^, 0.27 ± 0.11, *P* < 0.05; SW480^*IL-6R-ND2*^, 0.17 ± 0.06, *P* < 0.01) (Fig. 7C). Treatment of recombinant IL-6 did not suppress proliferation of SW480^*IL-6R-ND1*^ (Absorbance value of CCK-8; IL-6 0 ng/ml, 100 ± 8.0%; IL-6 0.01 ng/ml, 99 ± 10.1%) and SW480^*IL-6R-ND2*^ (Absorbance value of CCK-8; IL-6 0 ng/ml, 100 ± 4.65%; IL-6 0.01 ng/ml, 90 ± 4.4%), although that treatment suppressed proliferation of SW480^mock^ (Absorbance value of CCK-8; IL-6 0 ng/ml, 100 ± 9.3%; IL-6 0.01 ng/ml, 84 ± 7.6%; *P* < 0.01) (Fig. 7D). These results indicated that involvement of IL-6/IL-6R on proliferation of CRC cells in vitro.

## Discussion

In this study, we found that both OS and RFS in the TB^high^ group were significantly lower than those in the TB^low^ group and found that TB was one of the indications of poor prognosis in CRC patients. We could not show that a higher number of tumor buds was an independent poor prognostic factor in the multivariate analysis because of insufficient cases for statistical analysis in this study. Previously, a higher number of tumor buds has been demonstrated to be an independent poor prognostic factor in previous studies [3, 26], and tumor buds were demonstrated to have high metastatic potential [27]. Based on our and the previous findings, we assumed that CRC cells in the TB area may acquire a highly malignant phenotype, we examined the phenotype of CRC cells in the TB area.

A tumor bud has been defined as an isolated cancer cell or cluster comprising less than five cells [26]. We observed isolated CRC cells in the TB area (depth 5), as opposed to the clusters formed by many of those cells at depths 1–4. These results indicate that TB has been recognized as one of the morphological phenotypes of CRC cells in the invasive front, and TB would be involved in ITH in terms of morphological phenotype.

ITH has been shown to consist of subpopulations of cancer cells with different phenotypes [16]. These phenotypes are characterized by differences in gene expression (including protein expression), proliferation, metastatic potential, immunogenicity, and morphology [28]. Similarly, ITH has been shown to display diverse chemosensitivities. Chemotherapy diminishes the subpopulation with high sensitivity to chemotherapy so that the subpopulation with less sensitivity could be replaced. Selective pressure, including chemotherapy, metastatic cascade, and tumor–stromal interactions, has been proposed to generate cancer cells with a highly malignant phenotype [29–31]. Because cancer cells with a highly malignant phenotype have been shown to be involved in chemoresistance, selective pressure may play important roles in the acquisition of chemoresistance. Therefore, we examined the ITH of CRC in terms of the acquisition of chemoresistance in this study.

The acquisition of chemoresistance in cancer cells would be involved in the malignant phenotype of cancers. CRC cells with higher expression of TOPO1 have been shown to be more sensitive to TOPO1 inhibitors, such as irinotecan [19]. The decreasing expression of TOPO1 in CRC cells would imply resistance to irinotecan because irinotecan prevents the proliferation of CRC cells [19]. Previous reports demonstrated that CRC cells acquired strong chemoresistance [17, 32–35]. In this study, we demonstrated the heterogeneity of TOPO1 expression in CRC cells. Our IHC analysis revealed lower expression of TOPO1 in deeper CRC cells, especially CRC cells in the TB area (depth 5). These results indicated that the lowest expression of TOPO1 in CRC cells in the TB area would imply that those cells acquired strong chemoresistance. The expression of TOPO1 would be involved in ITH in terms of the acquisition of chemoresistance because the phenotype of CRC cells in the TB area differed from that at depths 1–4. We examined the expression of CD205 to elucidate the phenotype associated with malignancy in CRC cells in the TB area. CD205 is expressed mostly in antigen-presenting cells and occasionally in cancer cells [21, 22]. The expression of CD205 in CRC cells has been shown to decrease with malignancy [23], although the expression of CD205 in ovarian cancer cells has been shown to increase with malignancy [36]. In this study, we demonstrated that the expression of CD205 in CRC cells was lost in CRC cells in the TB area (depth 5) of all cases regardless of staining intensity in the invasive front without TB (depth 4). The expression of CD205 would be involved in ITH. In this study, we demonstrated that Ki-67 LI was lowered in CRC cells in the TB area indicating that proliferation of CRC cells would also be involved in ITH.

ITH was demonstrated to contribute to chemoresistance in CRC patients [37]. In this study, in terms of the acquisition of chemoresistance, very low expression of TOPO1 in CRC cells in the TB area may show strong chemoresistance. In terms of the malignant phenotype, loss of CD205 expression in CRC cells in the TB area may indicate a highly malignant phenotype. In terms of the proliferative potential, low Ki-67 LI of CRC cells in the TB area may indicate low proliferative activity. Taken together, the phenotypes of CRC cells in the TB area were clearly distinct from those at other depths. Because tumor–stromal interactions play an important role in altering the phenotype of CRC cells in the TB area, we performed a histological spatial analysis of tumor–stromal interactions between CRC cells and Mφs.

Tumor–stromal interactions are often observed in the TME. The TME consists of cancer cells and subsequently stromal cells, such as Mφs and fibroblasts [31]. Mφs have been demonstrated to interact with cancer cells in the TME [8, 9, 11], and often observed in the invasive front of CRC specimens [38, 39]. These findings indicated that Mφs might alter the phenotype of CRC cells in the invasive front. To demonstrate tumor–stromal interactions between CRC cells and Mφs in the invasive front, we focused on two histological findings: the number of Mφs per single CRC cell and the proximity between CRC cells and Mφs. Our CD68-IHC study revealed that the number of Mφs per single CRC cell in the TB area (depth 5) was 3.7 times higher than that in the invasive front without TB (depth 4) and 2.6 Mφs interacted with a single CRC cell in the TB area (depth 5). Similar results were obtained by CD163- and CD204-IHC. Interestingly, the number of CD206^+^ Mφs at depth 5 was very small compared to the number of CD163^+^ and CD204^+^ Mφs. Our results suggested that CD163^+^CD204^+^CD206^−^ Mφs were infiltrating in the TB area. Our histological spatial analysis revealed that the number of neighboring Mφs within 60 μm surrounding CRC cells in the TB area (depth 5) was three times higher than that in the invasive margin without TB (depth 4). The distance of 60 μm corresponds to the size of three Mφs because the size of Mφ is known to be approximately 20 μm. Taken together, both the number of Mφs per single CRC cell and the proximity between CRC cells and Mφs induced the interaction. Next, we examined the cytokines produced by Mφs that altered the phenotype of CRC cells in the TB area.

To elucidate the signaling pathway activated in CRC cells in the TB area, we examined the cytokines produced by Mφs. IL-6, IL-8, and CCL2 secreted from neighboring Mφs have been shown to alter the phenotype of cancer cells [8, 9, 40–46]. Our preliminary IHC studies of IL-6, IL-8, and CCL2 showed that IL-6 was successfully stained in Mφs. IL-6-derived Mφs might regulate the IL-6R/STAT3/miR-204-5p axis in CRC cells [9]. Our double-immunofluorescence staining revealed that many CD68^+^ Mφs and CD163^+^ Mφs produced IL-6 in the TB area (depth 5). Furthermore, our staining revealed that AE1/AE3-positive CRC cells in the TB area (depth 5) were positive for pSTAT3, indicating that the IL-6R/STAT3 signaling pathway may be upregulated in CRC cells by neighboring Mφs. These results suggested that IL-6 derived from neighboring Mφs activates CRC cells in the TB area via the IL-6R/STAT3 signaling pathway.

Although the action of irinotecan on CRC cells was demonstrated to be impaired by the activation of STAT3 [47], it is not known whether the nuclear translocation of pSTAT3 in CRC cells is directly involved in the decrease in TOPO1 expression in CRC cells. In future studies, we would like to show that TOPO1 expression is involved in activating the IL-6R/STAT3 signaling pathway in CRC cells in the TB area. Similarly, we would like to show that CD205 expression is involved in activating the IL-6R/STAT3 signaling pathway in CRC cells. Our data showed that Ki-67 LI was lowered in CRC cells in the TB area, and that IL-6/IL-6R suppressed proliferation of SW480 with high-expression of *IL-6R*. In the previous study, *CHP2*, which regulates cell proliferation, was downregulated in the TB area micro-dissected from CRC specimens [48]. Previous studies also demonstrated that Ki-67 LIs in the TB area were lower than other area (depth 1–4) [49, 50]. Thus, IL-6/IL-6R may suppress cell proliferation of CRC cells, especially in tumor buds. Contrary to our results, IL-6/IL-6R was shown to promote cell proliferation of CRC cells [51, 52], we will further investigate the molecular mechanism of proliferative activity of CRC cells at each depth.

## Conclusions

Our data are summarized as follows. A higher number of tumor buds contributed to poor prognosis in colorectal cancer patients. The phenotypes of colorectal cancer cells in the tumor budding area showed low-expression of topoisomerase 1, CD205, and Ki-67, and many neighboring macrophages altered the phenotype of colorectal cancer cells in the tumor budding area. This evidence may explain the intratumoral heterogeneity in terms of morphology, the acquisition of chemoresistance, and the malignant phenotype in colorectal cancer specimens. We found that the mechanism underlying this evidence would be the tumor–stromal interaction between colorectal cancer cells and macrophages in the tumor budding area via the interleukin-6 receptor/STAT3 signaling pathway.

### Supplementary Information

**Additional file 1: Figure S1.** IL-6 was secreted from CD163-positive macrophages at depth 5. **A** Double immunofluorescence of CD163 and IL-6. Nuclear staining was performed with DAPI. The dashed-line rectangle at depth 5 is magnified in Fig. 6B–D. **B** CD68-positive macrophages were labeled with TRITC red. **C** IL-6-positive cells were labeled with FITC green. **D** The merged yellow was observed at depth 5.

**Additional file 2: Supplemental Table S1.** Clinicopathological characteristics in 100 cases of colorectal cancer.

**Additional file 3: Supplemental Table S2.** Univariate and multivariative analyses for overall survival and relapse-free survival.

## Data Availability

Is available upon request from the corresponding author.

## Abbreviations

CCL2: C-C motif chemokine ligand 2
CRC: Colorectal cancer
EMT: Epithelial–mesenchymal transition
HE: Hematoxylin–eosin
IHC: Immunohistochemistry
IL-6: Interleukin-6
Mφ: Macrophage
OS: Overall survival
pSTAT3: Phospho-signal transducer and activator of transcription 3
RFS: Relapse-free survival
TB: Tumor budding
TOPO1: Topoisomerase 1
