# Supplementary figures and images for: Neighboring macrophage-induced alteration in the phenotype of colorectal cancer cells in the tumor budding area
Source: Cancer Cell Int. 2024 Mar 14;24:107. doi: 10.1186/s12935-024-03292-7 (PMC10938821; doi:10.1186/s12935-024-03292-7)

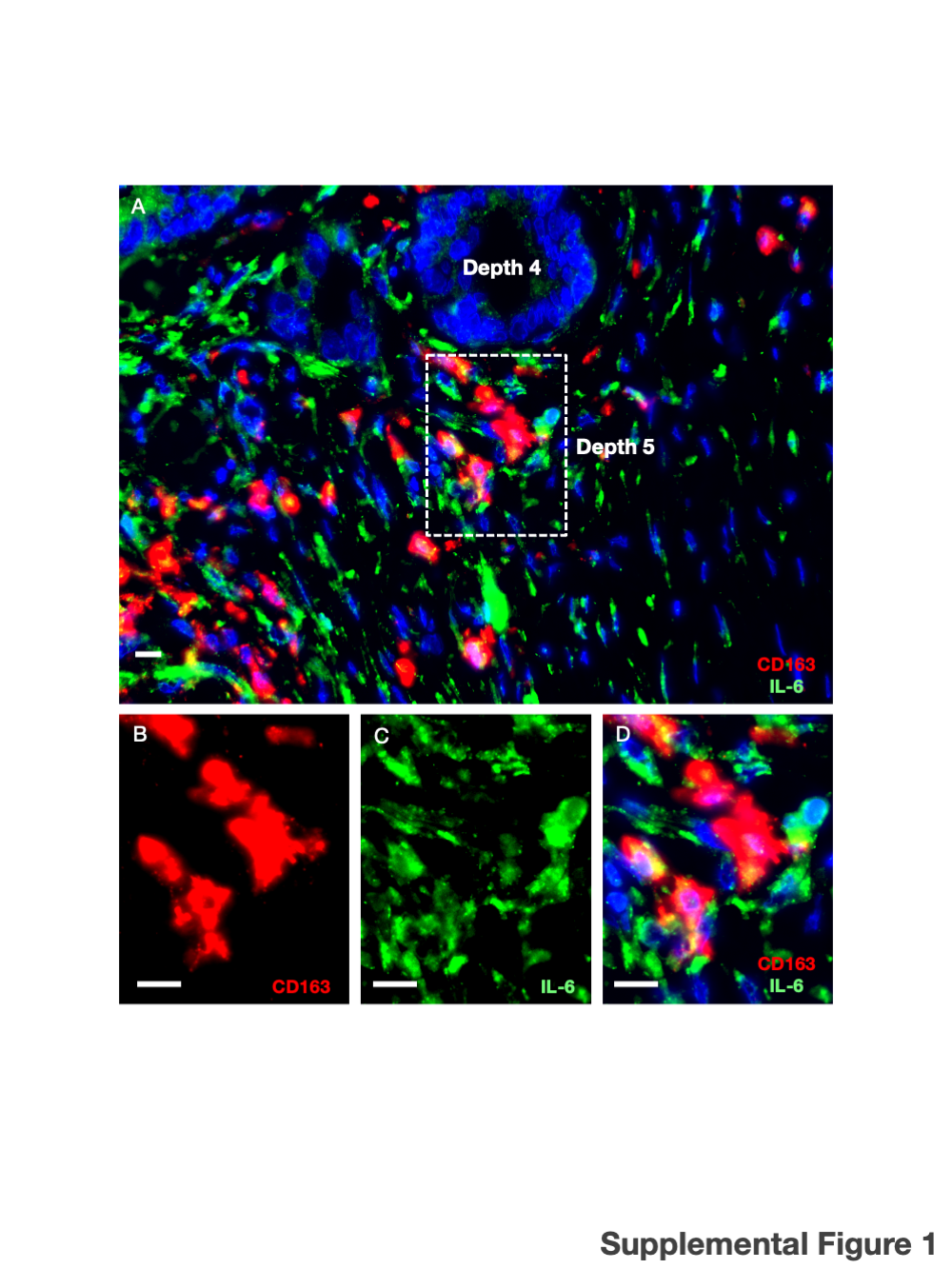

Supplement: Supplementary file 1 — Additional file 1: Figure S1. IL-6 was secreted from CD163-positive macrophages at depth 5. A Double immunofluorescence of CD163 and IL-6. Nuclear staining was performed with DAPI. The dashed-line rectangle at depth 5 is magnified in Fig. 6B–D. B CD68-positive macrophages were labeled with TRITC red. C IL-6-positive cells were labeled with FITC green. D The merged yellow was observed at depth 5. [file 12935_2024_3292_MOESM1_ESM.tiff]
