# Supplementary material for: Neighboring macrophage-induced alteration in the phenotype of colorectal cancer cells in the tumor budding area
Source: Cancer Cell Int. 2024 Mar 14;24:107. doi: 10.1186/s12935-024-03292-7 (PMC10938821; doi:10.1186/s12935-024-03292-7)
Supplement: Supplementary file 2 [file 12935_2024_3292_MOESM2_ESM.docx]

Table S1. Clinicopathological characteristics in 100 cases of colorectal cancer

Parameters	Number	TB low group	TB high group
Cases	100	61	39
Gender			
Male	60	39	21
Female	40	22	18
Age, years			
Mean±SD (range)		71.9±9.8(38-95)	68.9±12.4 (42-90)
Localization			
Right colon	53	38	15
Left colon	23	11	12
Rectum	24	12	12
Type			
Type 0	8	6	2
Type 1	11	5	6
Type 2	78	49	29
Type 3	1	0	1
Type 4	0	0	0
Tumor size, mm			
Mean±SD (range)		42.6±19.7 (10-110)	41.6±17.9 (6-83)
Histologic differentiation			
Pap, Well, Moderate	97	58	39
Others	3	3	0
Lymphatic invasion			
Positive	57	30	27
Negative	42	30	12
Vascular invasion			
Positive	21	11	10
Negative	78	49	29
Tumor budding (TB)			
Mean±SD (range)		5.9±4.1 (0-13)	27.9±15.7 (14-92)
Tumor depth/UICC			
T1	9	7	2
T2	18	11	7
T3	47	23	15
T4	26	11	15
Lymph node metastasis/UICC			
N0	51	34	17
N1	32	18	14
N2	17	9	8
Distant metastasis/UICC			
M0	78	51	27
M1	22	10	12
Stage/UICC			
Stage I	22	14	8
Stage II	25	19	6
Stage III	31	18	13
Stage IV	22	10	12
