# Supplementary material for: Neighboring macrophage-induced alteration in the phenotype of colorectal cancer cells in the tumor budding area
Source: Cancer Cell Int. 2024 Mar 14;24:107. doi: 10.1186/s12935-024-03292-7 (PMC10938821; doi:10.1186/s12935-024-03292-7)
Supplement: Supplementary file 3 [file 12935_2024_3292_MOESM3_ESM.docx]

Table S2. Univariate and multivariative analysis for overall survival

Parameters	Overall survival					
	Univariate analysis			Multivariate analysis		
	HR	(95% CI)	*P* value	HR	(95% CI)	*P* value
Gender：Male (baseline Female)	1.097	(0.539-2.324)	0.8013			
Age ≥70 (years)	1.326	(0.651-2.810)	0.4403			
Localization：Left colon, Rectum (baseline Right colon)	1.433	(0.707-2.954)	0.3174			
Tumor size ≥ 40 (mm)	1.780	(0.859-3.948)	0.1231			
Lymphatic invasion positivity	3.083	(1.397-7.759)	0.0044	1.288	(0.551-3.439)	0.5761
Vascular invasion positivity	1.957	(0.853-4.130)	0.1084			
Tumor budding ≥ 14 (high)	2.226	(1.097-4.642)	0.0267	1.583	(0.776-3.322)	0.2074
Tumor depth (UICC)：T3, T4 (baseline T1, T2)	2.982	(1.165-10.09)	0.0205			
Lymph node metastasis (UICC)：	4.608	(2.087-11.60)	<0.0001			
